# Impact of Soil Fertilized with Biomass Ash on Depth-Related Variability of Culturable Bacterial Diversity and Selected Physicochemical Parameters in Spring Barley Cultivation

**DOI:** 10.3390/ijerph192113721

**Published:** 2022-10-22

**Authors:** Miłosz Pastuszczak, Jadwiga Stanek-Tarkowska, Miroslava Kačániová

**Affiliations:** 1 Department of Soil Science, Environmental Chemistry and Hydrology, University of Rzeszow, 35-601 Rzeszów, Poland; 2 Department of Bioenergetics, Food Analysis and Microbiology, Institute of Food and Nutrition Technology, University of Rzeszow, 35-959 Rzeszow, Poland; 3 Faculty of Horticulture and Landscape Engineering, Institute of Horticulture, Slovak University of Agriculture, 94976 Nitra, Slovakia

**Keywords:** biomass ash, soil moisture, bulk density (BD), fertilization, soil bacteria, culturable microbial diversity, MALDI-TOF MS Biotyper

## Abstract

This study investigated the effect of different doses of fertilization with biomass combustion ash (*Salix viminalis* L. willow) on changes in the biological, chemical, and physical properties of soil. The experiment was carried out on podzolic and chernozem soils in a one-way field experiment (fertilization dose: control (without fertilization), NPK (nitrogen (N), phosphorus (P) and potassium (K)), 100, 200, 300, 400, 500 kg K_2_O ha^−1^). The biomass ash was characterized by a pH value of 12.83 ± 0.68 and a high content of macronutrients. The samples were collected from 0–5, 10–15, and 20–25 cm soil layers under the cultivation of spring barley (*Hordeum vulgare* L) cv. Planet in April and August 2021. Mass spectrometry (MALDI-TOF MS) was used for microbiological analyses, which revealed the presence of 53 culturable species from 11 genera: *Bacillus, Pseudomonas, Paenibacillus, Lysinibacillus, Pseudarthrobacter, Arthrobacter, Staphylococcus, Paenarthrobacter, Micrococcus, Rhodococcus*, and *Flavobacterium.* The podzolic and chernozem soils exhibited the presence of 28 and 44 culturable species, respectively. The study showed an increase in the number of microorganisms in the top layer of the soil profile. However, the number of bacteria decreased at the depths of 10–15 cm and 20–25 cm. With depth, the bulk density (BD) and moisture increased.

## 1. Introduction

Soil microorganisms from terrestrial habitats are key determinants of ecosystem services, e.g., nutrient cycling [1], stabilization of soil organic matter, and sequestration of carbon [2,3]. Bacterial communities play an important role in soil, contributing to such processes as organic matter turnover and nutrient cycling. Since the physicochemical properties of soil, i.e., pH, temperature, light availability, moisture and bulk density (BD), change with depth, the number of bacteria changes due to their ecological requirements. Investigations of the distribution of the microbiome in the soil profile [4] showed considerable variability with depth in terms of both the composition and the functional profiles of the community. Microorganisms colonize the entire soil profile and play an important role in nutrient cycling and soil formation. Data reported in several studies [5,6,7] indicate considerable differences in the bacterial diversity and composition between soil layers. However, little is known about vertical changes in soil bacterial communities occurring along depth gradients. Bacteria, fungi, archaea, actinomycetes and algae are the basic soil microorganisms, and bacteria are the most numerous group [8].

As indicated by Feng (2019) [6], soil depth is an important factor in the structure of environmental gradients determining the complexity and diversity of bacterial communities. The vertical spatial heterogeneity of the soil forces microorganisms to search for habitats selectively, which results in evident changes in the diversity and relative abundance of bacteria throughout the entire soil profile [6]. Soil depth represents a strong physiochemical gradient that greatly affects soil-dwelling microorganisms. Soil depth is an important factor affecting the microbiome composition. Significantly higher diversity and numbers of microorganisms are found in the nutrient-rich top layer of the soil, but they then decrease toward the less nutrient-rich bottom layer [9]. Mundr et al. (2021) [9] concluded that different groups of microorganisms are adapted to different soil layers, and the level of interactions between them varies with depth.

Most studies of bacterial communities have analyzed various soil cultivation systems with the classic mineral or organic fertilization schemes [10,11,12,13,14,15,16]. There are relatively few investigations of the application of biomass combustion ash as a fertilizer and its effect on the presence and growth of microorganisms in soils [17,18,19].

Various types of waste containing many valuable components are increasingly being used to fertilize soils and crops [17,18]. Biomass combustion ashes, which contain multiple plant-beneficial nutrients and can be used in plant cultivation, are this type of waste. Fertilization with ashes can also contribute to the improvement of the chemical and biological properties of soils, mainly by increasing the pH value, especially in acidic soils. Studies on the effect of wood ash on soil bacteria indicate its beneficial role in the soil-forming process [20,21,22,23,24,25,26]. Bang-Andreasen (2021, 2017) [17,18] showed a significant increase in the pH of forest soil and considerable changes in the composition of bacterial communities after the application of high doses of ash. Soils are regarded as one of the most diverse microbial habitats due to their high physical, chemical and biological heterogeneity. The present study focused on the distribution and composition of culturable bacterial species in the 0–25 cm soil profile. The main aim of the study was to determine changes in the pH, bulk density, moisture, and diversity of culturable bacteria in two types of soil (podzolic and chernozem) under spring barley cultivation and fertilization with various doses of woodchip combustion ash.

## 2. Materials and Methods

### 2.1. Experiment Model

In 2018, the experiment was established on a private farm on two types of soil, podzolic (P) and chernozem (C), with silty-clay graining (the soils were divided into granulometric subgroups according to the recommendations of the United States Department of Agriculture (USDA) [27] in Korzenica (Podkarpackie Province), GPS coordinates: 500.02′.16.3 N, 220.55′.06.4 E). A randomized block method (approximately 162 m^2^ in each block) in triplicate was used to set up the one-way field experiment. This type of experiment is commonly used in field experiments [28]. The main aim of the research was to use different doses of a “fertilizer”, i.e., ash from biomass combustion (*Salix viminalis* L. willow). Ash from biomass combustion was biologically pure, as the combustion temperature was between 1000 and 1200 °C. The recommended temperature was > 800 °C [29]. The doses used were as follows: control (without fertilization); NPK K_2_O in mineral fertilizers; 100 kg K_2_O ha^−1^ in ash (0.5 t ha^−1^ of ash in bulk weight); 200 kg K_2_O ha^−1^ in ash (1.0 t ha^−1^ of ash in bulk weight); 300 kg K_2_O ha^−1^ in ash (1.5 t ha^−1^ of ash in bulk weight); 400 kg K_2_O ha^−1^ in ash (2.0 t ha^−1^ of ash in bulk weight); and 500 kg K_2_O ha^−1^ in ash (2.5 t ha^−1^ of ash in bulk weight). The biomass ash doses were balanced by the amount of potassium introduced into the soil. In all variants of the experiment, constant mineral fertilization with nitrogen (81.3 kg N ha^−1^) and phosphorus (34 kg P ha^−1^) was applied. The obtained results were compared with the control soil (without fertilization) and with the soil on which classic mineral NPK fertilization was applied. The plant selected for the study was spring barley (*Hordeum vulgare* L) cv. Plants were cultivated in both soils.

The fertilizer was applied before sowing. Table 1 shows the fertilizer doses and application dates.

In the experiment, biomass ash was used to fertilize spring barley growing on podzolic and chernozem soils. Its composition is shown in Table 1.

### 2.2. Soil Sampling and Preparation for Analysis

Soil from the arable layer was collected in triplicate in April and August 2021 at the depths of 0–5, 10–15, and 20–25 cm. After drying and sieving through a 2 mm mesh, the physicochemical parameters of the soil samples were identified. An HI-4221 pH meter (Hanna Instruments, Nusfalau, Romania) was used to measure soil reaction with the potentiometric technique at a soil-to-solution ratio of 1:2.5. After the soil samples were dried to constant weight, the soil moisture was determined using the gravimetric technique. Additionally, the bulk density (BD) was measured.

### 2.3. Statistic Analysis

STATISTICA 13.3 (StatSoft, Tulsa, OK, USA) was used to examine the effect of biomass combustion ashes on the physicochemical parameters of the podzolic and chernozem soils. To identify homogenous groups (*p* < 0.05), a one-way analysis of variance (ANOVA) was conducted with a Tukey HSD multiple comparison test.

### 2.4. Microbiological Analysis

#### 2.4.1. Soil Preparation for Microbial Analyses

Before the microbiological analysis, an extra analysis of clean ash was performed by agar assay on plate count agar—PCA (BioMaxima SA, Lublin, Poland) medium, and the ash was shown to be microbiologically clean (no bacterial colonies were found).

In April (after eight months from fertilization) and August 2021(after 12 months from fertilization), soil samples (each 100 g in triplicate) were collected into sterile bags from the depths of 0–5, 10–15, and 20–25 cm in the experimental plots. A combined 300 g sample was taken from each plot. After sampling in the field, immediate analyses were made in the laboratory according to the recommended methodology. The soil was sampled twice in April and August 2021. The total number of microorganisms was determined with the plate count method, and the microorganisms were identified using a MALDI TOF mass spectrometer. For determination of the number of colony-forming units, the PCA medium was prepared according to the manufacturer’s instructions. This medium is used in the standard method to calculate the total number of culturable microorganisms. One gram of soil from the combined samples was weighed into sterile plastic test tubes together with 9 mL of distilled water and vortexed (Ohaus, Nänikon, Switzerland) for five minutes at 1500 rpm. Then, 100 µL of 10^−2^ and 10^−3^ serial dilutions were prepared and inoculated into Petri plates. The dishes were incubated at 30 °C for 48 h. Following the incubation, the colonies were counted, and the number of microorganisms in 1 g of soil was estimated [19].

#### 2.4.2. Mass Spectrometry Isolate Identification

The MALDI-TOF MS Biotyper instrument, which is appropriate for soil bacteria, was used in this research. Mass spectrometry was applied to identify bacteria isolated from the soil in the present study. Bacteria isolated from soils were identified in the current investigation using mass spectrometry. The study used a database which was built on the basis of distinct bacterial species that were isolated from the soil and then identified using molecular techniques [30] to analyze the various bacterial species. The MALDI-TOF MS Biotyper analysis sample was made in accordance with the extraction method recommended by the manufacturer (Bruker Daltonik, Bremen, Germany).

The sample was centrifuged at 13.000 rpm for two minutes after being mixed 10 times. The pellets were repeatedly centrifuged while the supernatant was discarded. The pellets were combined with 10 µL of 70% formic acid (*v*/*v*) (Sigma-Aldrich, Saint Louis, MO, USA) and the same volume of acetonitrile (Sigma-Aldrich, Saint Louis, MO, USA) after the supernatant was removed. The mixture was centrifuged once more, dyed with 1 L of the supernatant, and allowed to air dry at room temperature on a polished steel target plate. Each sample was supplemented with 1 µL of the MALDI matrix, which is a saturated solution of cyano-4-hydroxycinnamic acid (HCCA; Bruker Daltonik, Bremen, Germany), in 50% acetonitrile with 2.5% trifluoroacetic acid (Sigma-Aldrich, Saint Louis, MO, USA).

A Microflex LT MALDI-TOF mass spectrometer (Bruker Daltonik, Bremen, Germany) operating in a linearly positive mode in the mass range of 2000–20.000 Da produced the mass spectrometry data automatically. The Bruker bacterial standard was used to calibrate the apparatus. MALDI Biotyper 3.0 software was used to process the spectrometric results (Bruker Daltonik, Bremen, Germany). The following identification criteria were applied: a score between 1700 and 1999 indicated probable identification at the genus level, a score between 2000 and 2299 indicated safe genus identification with probable species identification, and a score between 2300 and 3000 indicated highly probable identification at the species level.

## 3. Results and Discussion

### 3.1. Effect of Weather Conditions on Soil Moisture

**Figure 1:**
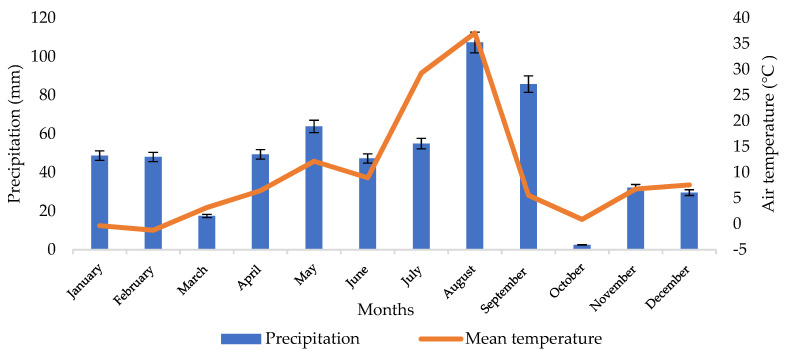
Data on weather conditions in 2021 provided by the meteorological Station of the University of Rzeszow.

Weather conditions are an important determinant of the growth of soil microorganisms. Figure 1 shows the weather conditions recorded in 2021, with the mean temperature of the coldest (February) and warmest (August) months of −1.2 °C and 21.6 °C, respectively. The lowest precipitation rate was recorded in October (2.5 mm), and the highest values of the parameter were noted in August (107.4 mm) and September (85.8 mm). The mean annual precipitation rate was 587.4 mm. Such weather conditions are favorable for the cultivation of spring barley. Its seeds are sown from 20 to 30 March at positive temperatures prevailing in Podkarpacie in this period.

Soil moisture is one of the most important determinants of soil physicochemical and biological properties [10,11]. It is associated with the type, granulometric composition, and water retention properties of soil, agrotechnical treatments, precipitation and air temperature.

An analysis of weather conditions in 2021 indicates that the highest monthly precipitation total was recorded in August, which suggests that the moisture content of the podzolic soil and the chernozem soil should be higher than in April. However, the high air temperature, which was 36 °C in August, caused intensive evaporation of the soil, which was without vegetation (the soil material was collected after harvesting the plants).

In the 0–5, 10–15 cm layer, the podzolic soil showed higher moisture content in April than in August in all variants of the experiment. An increase in moisture content compared to the control was recorded in all variants of the experiment. However, the highest value recorded for moisture content (37.25% *v/v*) was determined in the P5 variant fertilized with ash at 500 kg ha^−1^ at a depth of 10–15 cm. The highest precipitation was in August, but the air temperature was also high, so the moisture content in the 0–5 and 10–15 cm layers was lower due to intensive evaporation. In the 20–25 cm layer in Table 2, there is a noticeable increase in soil moisture relative to the top layers.

Chernozem soil reacted differently to weather conditions where the moisture content increased with depth (Table 3) at selected depths in the experiment, both in April and August. The moisture contents indicate that the application of bio-mass ash significantly increased the moisture content in the podzolic soil as well as the chernozem soil.

Pereira et al. [31] also studied the effect of ash on soil moisture content and received similar results. As the dose of ash increases, soil moisture content increases.

Statistical analysis showed significant differences at an application dose of 200 kgha^−1^ at a depth of 20–25 cm for both soils investigated.

### 3.2. Bulk Density

It is very important to determine the bulk density (BD), which is measured to characterize the compactness of soils in response to land use and fertilization [28]. Determination of this parameter reveals the size of infiltration and, hence, the water capacity and soil porosity, limitation of plant rooting, soil microbial activity, and the availability of soil air and, consequently, the availability of nutrients. An increase in bulk density entails a reduction in the size of macropores, an increase in the size of meso- and micropores, and consequent changes affecting the hydraulic conductivity parameter [32]. An increase in the bulk density of soil may indicate its degradation, i.e., deterioration of soil water retention, decreased oxygenation, and biological properties of soils [33,34,35]. Increasing bulk density not only induces changes in the pore size distribution, but also affects the soil’s ability to contract and conduct water [36,37,38,39], which adversely affects plants and soil microorganisms.

The results of a study conducted by Bonfim-Silva et al. (2022) [40] show that ash from biomass combustion causes a decrease in BD values in soil, which may have a beneficial effect on soil bacteria.

**Figure 2:**
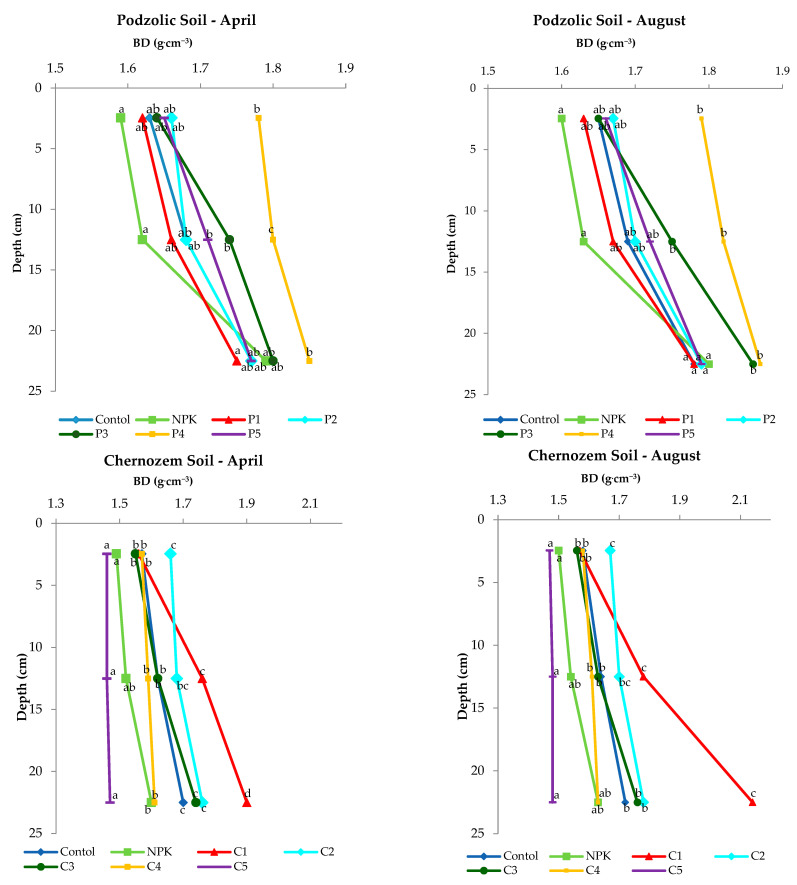
Changes in soil bulk density (BD) with depth in the experimental variants of podzolic and chernozem soils.

As reported by Wang (2020) [41], the lower the BD value, the greater the water permeability; this indicates better conditions for plant root development and bacterial growth. The analysis of the values obtained in the experiment (Figure 2) revealed an increase in BD with depth in both soils, which is a normal phenomenon. The application of ash from biomass combustion did not significantly increase BD values. Although the variants P3, P4 and C1 and C2 showed an increase in BD values, the application of the rate (500 kgha^−1^) on black soil affected the decrease in BD values compared to the other variants of the experiment. The lower BD values determined in April were related to agrotechnical operations and preparation of the soil in March for sowing spring barley. However, an insignificant increase in the BD value was found in both soils at a depth of 20 to 25 cm in August, which was related to the natural subsidence of the soil.

Statistical analysis showed no significant differences in the 0–5 cm topsoil between doses, while it showed significant differences at doses of 300, 400, 500 kgha^−1^ at depths of 10–15 cm and 20–25 cm for chernozem. However, in the case of chernozem soil, the 500 kgha^−1^ application dose, compared to the control and NPK, was statistically significant in April as well as in August. Statistically significant differences were found on chernozem soil when a dose of 100 kgha^−1^ (C1) and 500 kgha^−1^ (C5) was applied.

### 3.3. Soil pH

**Figure 3:**
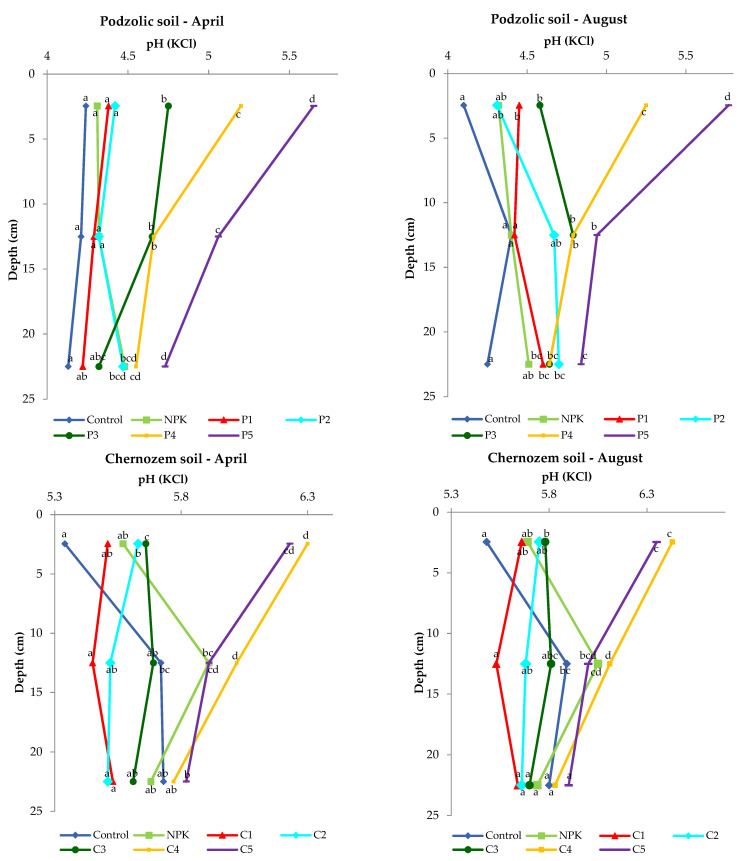
Differences in soil reaction upon the application of different ash doses.

As reported by Bloom (2012) [42], the soil pH is determined by the mineral composition and weathering of the soil parent material. For instance, soil acidification in moist environments persists for a long time, as the weathering products are washed away along with the lateral or downward movement of water through the soil. In turn, soil weathering and leaching in dry environments are less intense, and the soil pH is often neutral or alkaline. The pH is one of the most important parameters measured in soil, as it determines the life of microorganisms and plants. The biomass combustion ash used in the experiment had an alkaline pH of 12.82. The increase in soil pH induced by the application of the biomass combustion ash in each variant was significant and depended on the dose. In August, the pH of the podzolic soil was 4.1 in the control sample and 5.77 in the 0–5 cm layer in variant P5. Nevertheless, even a slight increase in pH may exert an effect on soil microorganisms (Figure 3). The results reported by Paul (2014) [43] and Bang-Andreasen (2017) [18] indicate the deacidifying properties of biomass ash. Similarly, the present study confirmed that the high dose of ash exerted a deacidifying effect on the 0–15 cm soil layer.

Statistical analysis showed significant differences in the 0–5 cm layer for the podzolic soil in April and in August for variants P3 to P5. For chernozem soil, statistically significant differences were found for variants C2–C5 in the top layer in April, while in August, statistically significant differences were found in the same layer for variants C3–C5.

In comparison with the podzolic soil, the chernozem control variant was characterized by a one-unit higher pH value in both April and August. The application of the different doses of the biomass combustion ash resulted in an increase in the reaction in the 0–5 cm layer and a slight decline in the value with depth.

In all the ash-fertilized variants, the soil pH was higher than its value in the control and NPK variants.

The presented results clearly show that the application of the ash doses of 400 and 500 kgha^−1^ in the case of both soils caused a significant increase in the surface layer in comparison with the control or NPK fertilization. Moreover, in the deeper layers, a similar trend was found, i.e., the application of biomass ash increased the pH of the examined soils.

### 3.4. Microorganisms

A wide range of edaphic factors, e.g., soil pH, nutrients, and carbon content, can determine the composition of soil microbial communities [44]. The activity and abundance of soil microorganisms are highly associated with many factors: soil type, crop type, temperature and moisture [45].

**Figure 4:**
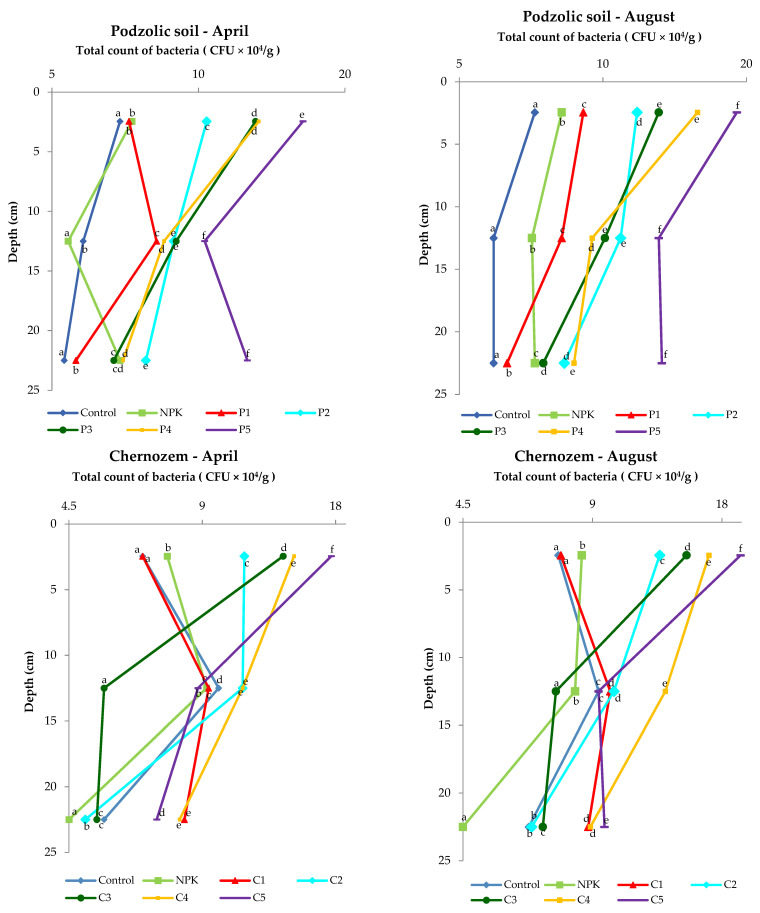
Differences in the number of bacteria (expressed in CFU × 10^4^/g of dry soil) in the 0–25 cm layer of podzolic and chernozem soils fertilized with different doses of the ash fertilizer.

**Figure 5:**
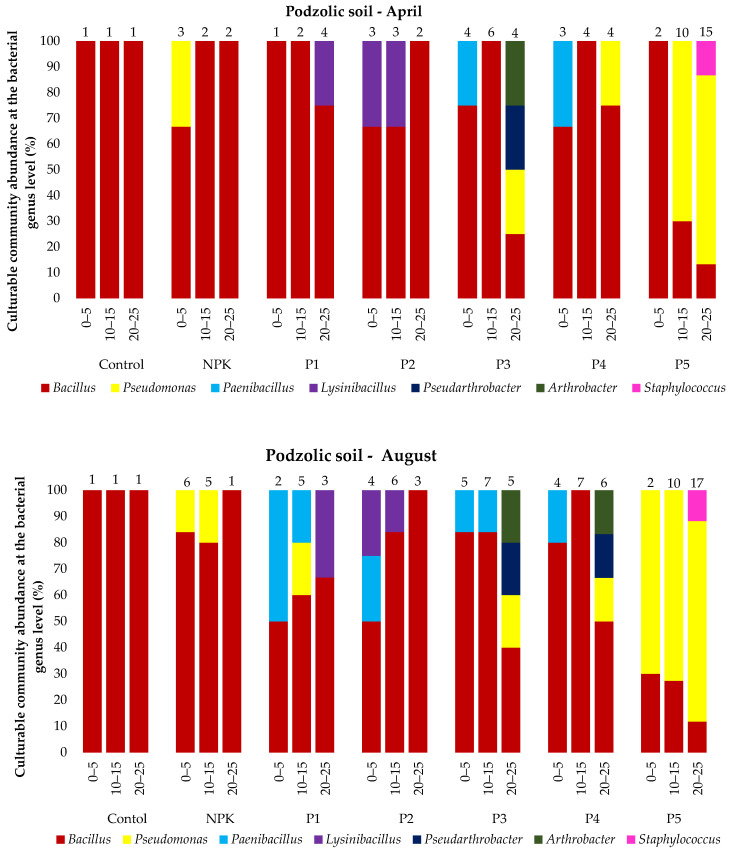

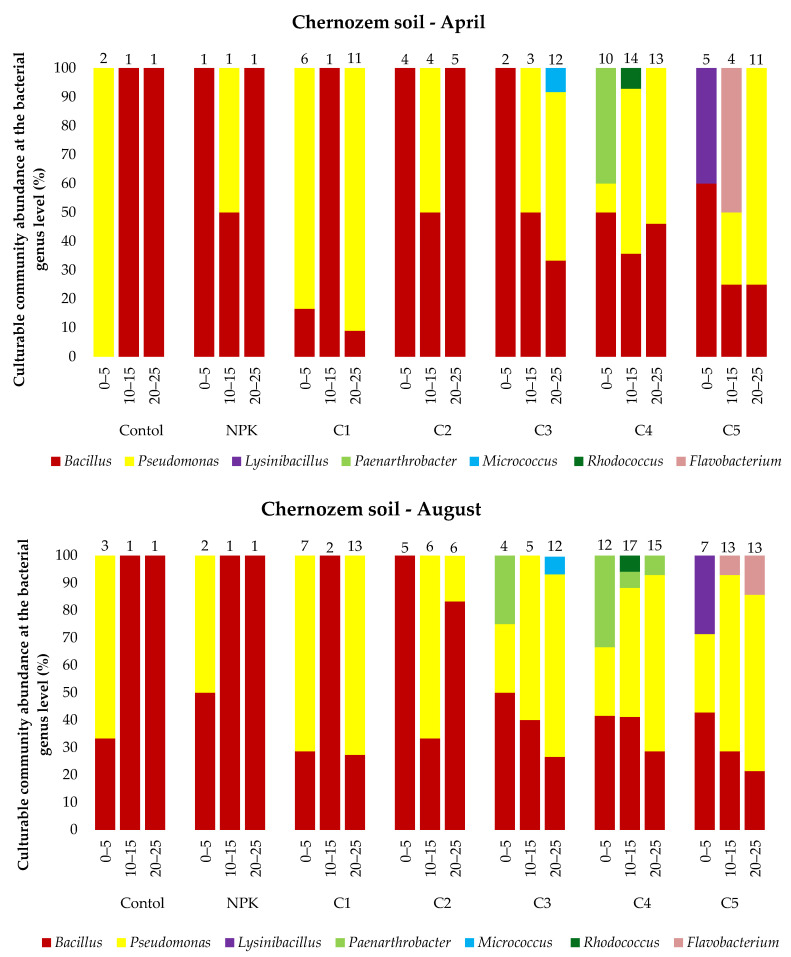
Culturable community abundance at the bacterial genus level (%) in the 0–25 cm layer of podzolic and chernozem soils in April and August 2021 after the application of different ash fertilizer doses; MALDI-TOF MS analysis. Numbers indicate the number of microorganisms identified.

The analysis of the microbial abundance revealed an increase in the number of bacteria in the two soil types depending on the ash dose (Figure 4). The list of bacterial species identified using MALDI-TOF MS is presented in the Supplementary Material Tables S1 and S2 and Figure 5. The smallest number of bacteria was found in the control variant of the podzolic soil in both April and August. The increase in the ash dose from 100 to 500 kgha^−1^ resulted in a significant increase in the number of microorganisms in both months. This relationship was also evident in the chernozem variants (Figure 4). The number of bacteria determined in both soils in April was lower than the number determined in August, which may have been caused by the higher soil moisture level (Table 2, Table 3) as a result of the maximum precipitation of 107.4 mm noted in August. The relationship between increased soil moisture and the number of bacteria was reported in other studies as well [46,47,48,49]. The analysis of the distribution of bacteria along the soil depth (Figure 4) in all the fertilization variants of the podzolic and chernozem soils demonstrated that the number of bacteria increased significantly to the depth of 0–5 cm and declined with depth. At a depth of 25 cm, the bacterial abundance was lower. The present study shows that BD and moisture content increase, but pH values did not change significantly along the soil profile depth. The profile-related differences in the analyzed soil parameters have an impact on the distribution of bacteria along soil depth [32,50,51]. Many other studies [7,52,53,54] confirmed the present observations of the greatest abundance of bacteria in the 10–15 cm layer. The 0–10 cm soil layer is unstable, as it is exposed to large fluctuations in moisture, temperature, pH, and BD associated with the impact of agrotechnical procedures.

Statistical analysis on the podzolic soil shows statistically significant differences between all fertilization variants at all tested depths. On the other hand, in the case of chernozem soil, there were no significant statistical differences in the topsoil for the control and C1 variants, while statistically significant differences were found for all variants at a depth of 20–25 cm. The present study demonstrated that fertilization with biomass combustion ash had a positive effect on the physicochemical properties of soil, contributing to an increase in the number of microorganisms. The beneficial effect of biomass combustion ashes was indicated in other studies [17,25,55,56,57], which reported an increase in the number and biodiversity of soil microorganisms induced by ash fertilization.

It was observed that even a slight reduction in the BD values in the topsoil layer had a positive effect on the number of microorganisms and thus improved the soil quality.

Clinical isolates can be identified using the MALDI-TOF MS Biotyper. Recent studies have demonstrated the suitability of the MALDI-TOF MS Biotyper for the identification of soil microorganisms as well. Mohammad et al. described the important role of microorganisms in the environment and how important it is to be able to rapidly identify them using a MALDI TOF MS Biotyper, which gives us answers to changes in the soil environment [58].

In total, 53 bacterial species were identified in all the variants of the podzolic and chernozem soils. They represented 11 genera, i.e., *Bacillus*, *Pseudomonas*, *Paenibacillus*, *Lysinibacillus, Pseudarthrobacter, Arthrobacter, Staphylococcus, Paenarthrobacter, Micrococcus, Rhodococcus*, and *Flavobacterium. Bacillus*, represented by 28 species, was the most common genus in all the variants of the podzolic soil. The ash-fertilized variants exhibited greater biodiversity and a higher number of identified microorganisms. The highest number of identified microorganisms was detected in variant P5, and the highest biodiversity was determined in variant P3, where *Pseudomonas, Paenibacillus, Lysinibacillus, Pseudarthrobacter, Arthrobacter* and *Staphylococcus* were identified. In turn, 44 species were identified in the chernozem samples. *Bacillus* species were identified in all the variants most frequently, but the variants fertilized with increasing ash doses exhibited a different species composition than in the podzolic soil, as they contained *Pseudomonas, Lysinibacillus, Paenarthrobacter, Micrococcus, Rhodococcus* and *Flavobacterium*. The greatest species diversity was detected in the chernozem variant C5 (500 kgha^−1^ ash). The majority of the chernozem variants exhibited a considerable number of *Pseudomonas* bacteria.

The culturable bacterial species diversity between the studied soil types indicates interactions between soil properties and bacteria [25,47]. In general, the variants fertilized with the different doses of ash were characterized by an increase in the number and biodiversity of identified microorganisms in comparison with the control variants and those fertilized with the traditional NPK fertilizer. This indicates a positive effect of ash on the species richness, diversity, and composition of the bacterial community [17,18,59]) and improvement of the physicochemical properties of the soil. As suggested by Tian (2021) [7], microorganisms colonize the entire soil profile and play an important role in nutrient cycling and soil formation. Recent studies have shown that the bacterial diversity and composition in different soil layers vary significantly. The bacterial variability in the present study was related to soil moisture, pH, and BD. Similar results were obtained by Shen (2013) [54].

The increase in the culturable abundance and variety of bacteria induced by the application of biomass combustion ash is probably associated with increased amounts of available nutrients. Ash contains many components, which are utilized as nutrients by soil bacteria [60].

Bang-Andreasen (2020) [61] reported that wood ash had a significant effect on the taxonomic and functional profile of agricultural and forest soils. Increased pH, electrical conductivity, dissolved organic carbon and phosphates were the most important physicochemical determinants of observed changes. The author also found that the addition of wood ash increased the relative abundance of the bacterial groups. The present study also showed an increase in the number of bacteria ascribed to the positive effect of ash fertilization.

Soil contains a wide range of microorganisms, including beneficial and potentially pathogenic species [62]. Bacteria may be beneficial, e.g., plant growth-promoting bacteria (PGPB), harmful (phytopathogens) or neutral in their interactions with plants [63]. PGPB can exert a favorable influence on plant growth and development in many different ways and in various environmental conditions. Their positive impact includes support of plant growth via increasing plant biomass, the elevation of plant mineral content (iron, phosphorus, and potassium), a constant supply of nitrogen to plants, elongation of roots and/or shoots, enhancement of seed germination, protection of plants against a wide range of phytopathogenic organisms, enhancement of plant tolerance to a wide range of environmental stresses, intensification of the production of useful secondary metabolites, and improvement of the overall plant nutrition status [64,65,66,67,68]. PGPB are classified as microorganisms exerting a positive effect on soil and plants. Due to their competition with bacterial communities present in the rhizosphere, PGPB are regarded as a tool for sustainable farming and a trend in future agriculture. The PGPB group comprises many genera that have been studied extensively for many years, i.e., *Acetobacter, Acinetobacter, Alcaligenes, Arthrobacter, Azoarcus, Azospirillum, Azotobacter, Bacillus, Beijerinckia, Burkholderia, Derxia, Enterobacter, Gluconacetobacter, Herbaspirillum, Klebsiella, Ochrobactrum, Pantoae, Paenarthrobacter, Pseudarthrobacter, Pseudomonas, Rhodococcus, Serratia, Stenotrophomonas, Zoogloea, Paenibacillus, Lysinibacillus* and *Staphylococcus* [69,70,71,72,73].

*Bacillus* species from the PGP (plant growth promoting) group were identified most frequently in the present study. These bacteria use multiple direct and indirect mechanisms to improve and enhance plant growth and productivity in various environmental conditions. They use distinctive direct mechanisms facilitating plant growth, e.g., nutrient dissolution and delivery, nitrogen fixation, production of siderophores and phytohormones, and modulation of plant hormone levels. The indirect mechanisms include the production of exopolysaccharides, hydrogen cyanide and lytic enzymes, the formation of biofilm, antibiosis, and induced systemic immunity [72,73,74,75,76,77,78]. As demonstrated by Tiwari (2019) [74], these mechanisms increase plant yields directly or indirectly.

Another large group identified in the present study was the genus *Pseudomonas* exhibiting special traits, i.e., efficient root colonization, production of osmolytes, polysaccharides, and phytohormones, production of specific enzymes, ability to adapt to stress, and positive interactions with other microbial communities [79].

*Paenibacillus* is another bacterial group identified in the ash-fertilized variants. As shown by Grady (2016) [80], this bacterial group is beneficial and useful in agriculture. The species serve many important functions, e.g., nitrogen fixation, phosphate solubilization, iron metabolism, phytohormone production, biocontrol, induced systemic resistance, and insecticidal and bactericidal activity through the production of various enzymes [80].

Another important bacterial group is *Lysinibacillus*. Ahsan (2021) [81] found that this group has the potential to combat pests, remediate heavy metal-contaminated environments, and increase crop yields. Since *Lysinibacillus* species produce spores, they can be considered a suitable agent to be used in microbiological products [81].

The *Pseudarthrobacter* group of beneficial bacteria is resistant to heavy metals and has the ability to promote plant growth [82]. Similarly, the *Rhodococcus* species identified in variant C4 is suitable for bioremediation of contaminated soils [83].

The podzolic soil variants P3 and P4 contained *Arthrobacter* bacteria. They are able to use both inorganic and organic substances as a substrate for metabolism, as reported by Krishnan (2016) [84], carrying out bioremediation activity and the removal of pollutants from soil and groundwater by live microorganisms mostly found in the rhizosphere. The development and yields of plants are benefited by several strains in this category. They are protected from abiotic stress [84].

Bacteria from the *Staphylococcus* group were found only in variant P5 of the podzolic soil. It has been reported in the literature that many *Staphylococcus* species are associated with plants and exert a significant positive effect on growth, stimulate plant growth in drought conditions, and reduce heavy metals to non-toxic compounds [85,86].

*Paenarthrobacter* strains have a potential biostimulatory capacity via the production of auxin or decomposition of amino acids. Under drought stress, they can improve plant growth through the promotion of the development and expansion of the root system in order to uptake the greatest amount of water and reduce stress perception in plants [87].

In contrast, *Micrococcus* strains were identified only in variant C3. They exhibit dual biocontrol potential. One of them is the direct inhibition of plant pathogen growth through the production of hydrogen cyanide and siderophore combined with specific enzymatic activities (cellulase, protease, and chitinase). The indirect mode involves the inhibition of plant pathogenic fungi through antifungal activity based on the production of secondary metabolites that have a toxic effect on pathogens [88].

The *Flavobacterium* strains identified in variant C5 were not directly associated with plant growth protection in the past. However, studies conducted by Manter (2010) and Sang and Kim (2012) [89,90] showed that their number in the rhizosphere was positively correlated with plant biomass, plant resistance to pathogens, and the stimulation of fruit ripening; hence, they can be classified as beneficial bacteria.

In field studies, the timing of sampling and the specific presentation of weather conditions are very important. The current study shows a significant impact on the physicochemical and microbiological parameters of soil material sampled in April compared to August. The higher monthly precipitation totals and air temperatures may have affected the diversity and abundance of microorganisms. Evaporation is most intense in the 0–5 cm topsoil layer, with evaporation decreasing with depth.

## 4. Conclusions

The present study has shown that fertilization of soils with biomass combustion ash improves their biological, chemical and physical qualities. Bacterial communities present in the soil play a very important role in their direct and indirect impact on soil quality and fertility. The experiment has demonstrated that the application of biomass combustion ash is responsible for strong vertical gradients of environmental parameters in the top parts of the soil profile, i.e., 0–25 cm. This is indicated by the increase in pH, BD, and moisture. However, the abundance of microorganisms decreased with depth. The application of the highest ash doses of 400 and 500 kg K_2_O ha^−1^ had the most beneficial effect on the physicochemical parameters studied and on the number and community of bacteria. Both the composition and abundance of culturable bacterial communities in the soil are directly related to soil depth and agrotechnical treatments. The bacterial composition varies depending on the type of soil. Such genera as *Bacillus* and *Pseudomonas*, which can be called cosmopolitan species, are common in soils. However, the presence of some bacterial species is soil specific. It is determined by the physicochemical properties of soils and cultivation and fertilization treatments.

In field experiments, analyses of soil material in terms of their biological, chemical, or physical properties and weather conditions, i.e., the amount of precipitation and air temperature, should be taken into account, as they have a significant impact on the final results.

## Figures and Tables

**Table 1:** Overview of the characteristics of ash from biomass combustion and applied fertilizer doses.

Composition of Biomass Ash Used in the Experiment for Fertilization of Winter Oilseed Rape on Podzolic Soil and Chernozem						
pH H_2_O	EC µS·cm^−^^1^	Ca (mg kg^−^^1^)	K (mg kg^−^^1^)	Na (mg kg^−^^1^)		P (mg kg^−^^1^)
12.82	8.81	145.081	129.617	1452		9244
Fertilizers used in the two field experiments in 2018–2021 podzolic soil and chernozem						
Fertilizer-Trade Name		Amount of Pure Component in 100 kg of the Fertilizer		Dose (kg/L per 1 ha)		Date of Application
				Fertilizer	Pure Component	
Biomass combustion ash		1.63 %P (3,73 kg P), 19.4% K (23.37 kg K), 4.96% Mg (8.222 kg Mg)		Varied depending on the experimental variant		30 August 2018 29 August 2019 25 August 2020
Monoammonium phosphate (MAP) NH_4_H_2_PO_4_ (12%N-NH_4_, 52% P_2_O_5_, 22.7% P)		22.7 kg P		150	34	30 August 2018 (all variants) 29 August 2019 (all variants) 25 August 2020 (all variants)
		12 kg N			18	
Potassium salt (60%)		60 kg K		175	105	30 August 2018 (NPK variant only) 29 August 2019 (NPK variant only) 28 August 2020 (NPK variant only)
RSM ^®^ 32% N (aqueous solution of urea-ammonium nitrate, density 1.32 kg/dcm^3^)		42.2 kg N (32 × 1.32)		150	63.3	4 March 2019 10 March 2020 15 March 2021

**Table 2:** Podzolic soil—Moisture *v*/*v*%. Control (without fertilization), NPK K_2_O in mineral fertilizers, P1–100 kg K_2_O ha^−1^ in ash (0.5 t ha^−1^ of ash in bulk weight), P2–200 kg K_2_O ha^−1^ in ash (1.0 t ha^−1^ of ash in bulk weight), P3–300 kg K_2_O ha^−1^ in ash (1.5 t ha^−1^ of ash in bulk weight), P4–400 kg K_2_O ha^−1^ in ash (2.0 t ha^−1^ of ash in bulk weight), P5–500 kg K_2_O ha-1 in ash (2.5 t ha^−1^ of ash in bulk weight).

Depth (cm)	Control	NPK	P1	P2	P3	P4	P5
April							
0–5	28.1 ^a^	31.15 ^b^	33.01 ^bc^	34.31 ^cd^	34.76 ^cd^	35.91 ^d^	36.46 ^d^
10–15	29.05 ^a^	30.01 ^a^	31.12 ^a^	31.54 ^a^	35.94 ^b^	36.35 ^b^	37.25 ^b^
20–25	23.26 ^a^	24.12 ^ab^	25.69 ^ab^	26.18 ^bc^	28.9 ^cd^	30.14 ^d^	31.12 ^d^
August							
0–5	21.01 ^a^	21.05 ^a^	22.99 ^ab^	22.75 ^ab^	25.21 ^bc^	25.3 ^bc^	26.65 ^c^
10–15	26.78 ^a^	27.53 ^ab^	27.76 ^abc^	28.95 ^abc^	29.4 ^abc^	30.17 ^bc^	30.51 ^c^
20–25	28.03 ^a^	29.02 ^ab^	29.56 ^ab^	30.07 ^abc^	31.03 ^bc^	31.51 ^bc^	32.52 ^c^

Same lowercase are not statistically significant, different lowercase letters indicate significant differences between variants of experiment on the same depth by Tukey’s HSD post hoc test (*p* > 0.05).

**Table 3:** Chernozem soil—Moisture *v/v*%. Control (without fertilization), NPK K_2_O in mineral fertilizers, C1–100 kg K_2_O ha^−1^ in ash (0.5 t ha^−1^ of ash in bulk weight), C2–200 kg K_2_O ha^−1^ in ash (1.0 t ha^−1^ of ash in bulk weight), C3–300 kg K_2_O ha^−1^ in ash (1.5 t ha^−1^ of ash in bulk weight), C4–400 kg K_2_O ha^−1^ in ash (2.0 t ha^−1^ of ash in bulk weight), C5–500 kg K_2_O ha^−1^ in ash (2.5 t ha^−1^ of ash in bulk weight).

Depth (cm)	Control	NPK	C1	C2	C3	C4	C5
April							
0–5	31.46 ^a^	32.12 ^a^	32.71 ^a^	35.61 ^b^	36.39 ^b^	36.96 ^b^	37.74 ^b^
10–15	32.98 ^a^	33.1 ^a^	33.15 ^a^	37.12 ^b^	37.96 ^b^	38.52 ^b^	39.05 ^b^
20–25	33.25 ^a^	34.58 ^a^	35.98 ^ab^	37.75 ^bc^	38.08 ^bc^	39.12 ^c^	39.64 ^c^
August							
0–5	31.37 ^a^	32.4 ^ab^	32.8 ^abc^	34.51 ^bcd^	35.49 ^cd^	35.89 ^d^	36.57 ^d^
10–15	32.3 ^a^	33.48 ^a^	34.06 ^ab^	36.57 ^bc^	37.19 ^c^	37.46 ^c^	38.97 ^c^
20–25	33.09 ^a^	34.39 ^a^	35.56 ^ab^	37.67 ^bc^	38.59 ^c^	38.88 ^c^	39.52 ^c^

Same lowercase are not statistically significant, different lowercase letters indicate significant differences between variants of experiment on the same depth by Tukey’s HSD post hoc test (*p* > 0.05).

## Data Availability

The entire set of raw data presented in this study is available on request from the corresponding author, The data presented in this study are available in this article.

## Supplementary Materials

The following supporting information can be downloaded at: https://www.mdpi.com/article/10.3390/ijerph192113721/s1. Table S1: List of bacterial species identified using MALDI-TOF MS in the podzolic soils with a depth of 0–25 cm in April and August 2021 for the cultivation of spring barley. Control (without fertilization), NPK K_2_O in mineral fertilizers, P1–100 kg K_2_O ha^−1^ in ash (0.5 t ha^−1^ of ash in bulk weight), P2–200 kg K_2_O ha^−1^ in ash (1.0 t ha^−1^ of ash in bulk weight), P3–300 kg K_2_O ha^−1^ in ash (1.5 t ha^−1^ of ash in bulk weight), P4–400 kg K_2_O ha^−1^ in ash (2.0 t ha^−1^ of ash in bulk weight), P5–500 kg K_2_O ha^−1^ in ash (2.5 t ha^−1^ of ash in bulk weight), Table S2: List of bacterial species identified using MALDI-TOF MS in the chernozem soil with a depth of 0–25 cm in April and August 2021 for the cultivation of spring barley. Control (without fertilization), NPK K_2_O in mineral fertilizers, C1–100 kg K_2_O ha^−1^ in ash (0.5 t ha^−1^ of ash in bulk weight), C2–200 kg K_2_O ha^−1^ in ash (1.0 t ha^−1^ of ash in bulk weight), C3–300 kg K_2_O ha^−1^ in ash (1.5 t ha^−1^ of ash in bulk weight), C4–400 kg K_2_O ha^−1^ in ash (2.0 t ha^−1^ of ash in bulk weight), C5–500 kg K_2_O ha^−1^ in ash (2.5 t ha^−1^ of ash in bulk weight).
