# Supplementary material for: Impact of Soil Fertilized with Biomass Ash on Depth-Related Variability of Culturable Bacterial Diversity and Selected Physicochemical Parameters in Spring Barley Cultivation
Source: Int J Environ Res Public Health. 2022 Oct 22;19(21):13721. doi: 10.3390/ijerph192113721 (PMC9658306; doi:10.3390/ijerph192113721)
Supplement: Supplementary file 1 [file ijerph-19-13721-s001.zip › ijerph-1895337-supplementary.pdf]

Table S1. List of bacterial species identified using MALDI-TOF MS in the podzolic soils with a depth of 0 - 25 cm in April and August 2021 for the cultivation of spring barley. Control (without fertilization), NPK K_2O in mineral fertilizers, P1–100 kg K_2O ha^{-1} in ash (0.5 t ha^{-1} of ash in bulk weight), P2–200 kg K_2O ha^{-1} in ash (1.0 t ha^{-1} of ash in bulk weight), P3–300 kg K2O ha^{-1} in ash (1.5 t ha^{-1} of ash in bulk weight), P4–400 kg K_2O ha^{-1} in ash (2.0 t ha^{-1} of ash in bulk weight), P5–500 kg K_2O ha^{-1} in ash (2.5 t ha^{-1} of ash in bulk weight).

Taxa	Podzolic soil																				
	April																				
	Control			NPK			P1			P2			P3			P4			P5		
	0-5	10-15	20-25	0-5	10-15	20-25	0-5	10-15	20-25	0-5	10-15	20-25	0-5	10-15	20-25	0-5	10-15	20-25	0-5	10-15	20-25
Arthrobacter globiformis															+						
Bacillus amyloliquefaciens ssp. *amyloliquefaciens*														+							
Bacillus amyloliquefaciens ssp. *plantarum*														+							
Bacillus cereus				+	+	+		+	+	+	+	+	+	+		+	+	+	+	+	+
Bacillus megaterium	+	+	+	+	+	+	+	+	+	+	+	+			+	+	+	+	+	+	
Bacillus mycoides									+					+			+				
Bacillus pseudomycoides																		+			
Bacillus thuringiensis													+	+			+			+	+
Bacillus weihenstephanensis													+	+							
Lysinibacillus fusiformis									+	+	+										
Paenibacillus amylolyticus													+								
Pseudarthrobacter oxydans															+	+					

Taxa	August																				
	Control			NPK			P1			P2			P3			P4			P5		
	0-5	10-15	20-25	0-5	10-15	20-25	0-5	10-15	20-25	0-5	10-15	20-25	0-5	10-15	20-25	0-5	10-15	20-25	0-5	10-15	20-25
Arthrobacter globiformis															+			+			
Bacillus amyloliquefaciens ssp. *amyloliquefaciens*														+			+				
Bacillus amyloliquefaciens ssp. *plantarum*														+			+				
Bacillus cereus				+	+	+		+	+	+	+	+	+	+		+	+	+	+	+	+
Bacillus megaterium	+	+	+	+	+		+	+	+	+	+	+			+	+	+	+	+	+	
Bacillus mycoides				+	+						+		+	+	+		+				
Bacillus pseudomycoides				+				+			+							+			
Bacillus thuringiensis											+		+	+		+	+			+	+
Bacillus weihenstephanensis				+	+								+	+		+	+				
Lysinibacillus fusiformis									+	+	+	+									
Paenibacillus amylolyticus								+		+			+	+							
Pseudarthrobacter oxydans															+			+			

Pseudomonas brassicacearum																				+	+																				+	+
Pseudomonas chlororaphis																		+		+	+							+	+												+	+
Pseudomonas chlororaphis ssp. *aurantiaca*																				+	+																				+	+
Pseudomonas chlororaphis ssp. *chlororaphis*																					+																					+
Pseudomonas corrugata																					+																					+
Pseudomonas frederiksbergensis				+											+						+				+	+										+			+			+
Pseudomonas fuscovaginae																					+																					+
Pseudomonas jessenii																				+																					+	
Pseudomonas kilonensis																					+																					+
Pseudomonas mandelii																				+																					+	+
Pseudomonas migulae																				+																					+	+
Pseudomonas rhodesiae																					+																					+
Pseudomonas thivervalensis																				+	+																				+	+
Pseudomonas vancouverensis																					+																					+
Staphylococcus aureus																					+																					+
Staphylococcus aureus ssp. *aureus*																					+																					+
**Total**	**1**	**1**	**1**	**3**	**2**	**2**	**1**	**2**	**4**	**3**	**3**	**2**	**4**	**6**	**4**	**3**	**4**	**4**	**2**	**10**	**15**	**1**	**1**	**1**	**6**	**5**	**1**	**2**	**5**	**3**	**4**	**6**	**3**	**5**	**7**	**5**	**4**	**7**	**6**	**2**	**10**	**17**

Table S2. List of bacterial species identified using MALDI-TOF MS in the chernozem soil with a depth of 0 - 25 cm in April and August 2021 for the cultivation of spring barley. Control (without fertilization), NPK K_2O in mineral fertilizers, C1–100 kg K_2O ha^{-1} in ash (0.5 t ha^{-1} of ash in bulk weight), C2–200 kg K_2O ha^{-1} in ash (1.0 t ha^{-1} of ash in bulk weight), C3–300 kg K2O ha^{-1} in ash (1.5 t ha^{-1} of ash in bulk weight), C4–400 kg K_2O ha^{-1} in ash (2.0 t ha^{-1} of ash in bulk weight), C5–500 kg K_2O ha^{-1} in ash (2.5 t ha^{-1} of ash in bulk weight).

Taxa	Chernozem soil																																									
	April																					August																				
	Control			NPK			C1			C2			C3			C4			C5			Control			NPK			C1			C2			C3			C4			C5		
	0-5	10-15	20-25	0-5	10-15	20-25	0-5	10-15	20-25	0-5	10-15	20-25	0-5	10-15	20-25	0-5	10-15	20-25	0-5	10-15	20-25	0-5	10-15	20-25	0-5	10-15	20-25	0-5	10-15	20-25	0-5	10-15	20-25	0-5	10-15	20-25	0-5	10-15	20-25	0-5	10-15	20-25
Bacillus cereus			+					+		+	+	+	+	+	+	+	+	+	+		+			+				+	+	+	+	+	+	+	+	+	+	+	+	+	+	+
Bacillus cytotoxicus						+												+																				+	+			
Bacillus megaterium		+		+	+				+	+	+	+			+	+			+	+	+	+	+		+	+	+	+	+	+	+	+	+			+	+	+	+	+	+	+
Bacillus mycoides										+						+	+														+						+	+				
Bacillus pseudomycoides												+			+			+			+												+			+			+		+	+
Bacillus simplex							+					+					+	+	+														+					+	+	+		
Bacillus thuringiensis										+			+	+	+	+	+	+													+			+	+	+	+	+	+			
Bacillus weihenstephanensis												+				+	+	+												+	+		+				+	+				
Flavobacterium aquidurense																				+																					+	+
Flavobacterium saccharophilum																				+																					+	+
Lysinibacillus fusiformis																			+																					+		
Lysinibacillus sphaericus																			+																	+						
Micrococcus luteus															+																					+						
Paenarthrobacter aurescens																+																					+					
Paenarthrobacter histidinolovorans																+																					+					
Paenarthrobacter ilicis																+																		+			+	+	+			
Paenarthrobacter nicotinovorans																+																					+					
Paenibacillus amylolyticus																																										
Pseudarthrobacter oxydans																																										
Pseudomonas agarici									+																					+			+									
Pseudomonas azotoformans							+																					+														
Pseudomonas brassicacearum									+						+		+	+			+									+						+	+	+	+		+	+
Pseudomonas brenneri																					+																				+	+

Pseudomonas caricapapayae																	+																					+	+			
Pseudomonas chlororaphis							+				+														+			+				+										
Pseudomonas chlororaphis ssp. *aurantiaca*									+									+												+									+			
Pseudomonas chlororaphis ssp. *chlororaphis*									+									+												+									+			
Pseudomonas corrugata							+										+				+							+				+			+			+			+	+
Pseudomonas extremorientalis	+																					+																				
Pseudomonas frederiksbergensis									+					+	+	+	+													+				+	+	+	+	+		+		
Pseudomonas fuscovaginae																																										
Pseudomonas gessardii															+																					+						
Pseudomonas graminis																	+																					+				
Pseudomonas jessenii									+						+			+												+						+			+			
Pseudomonas kilonensis									+								+	+												+						+	+	+	+			
Pseudomonas koreensis							+				+						+											+				+			+	+		+	+			
Pseudomonas libanensis	+																				+	+																			+	+
Pseudomonas mandelii									+																					+						+						
Pseudomonas migulae																					+																				+	+
Pseudomonas orientalis																	+																					+		+		
Pseudomonas protegens																					+																				+	+
Pseudomonas proteolytica							+								+													+								+						
Pseudomonas rhodesiae																					+																				+	+
Pseudomonas thivervalensis									+						+			+		+	+									+						+			+		+	+
Pseudomonas trivialis																																+										
Pseudomonas vancouverensis									+						+			+												+						+						
Rhodococcus erythropolis																	+																					+				
**Total**	**2**	**1**	**1**	**1**	**1**	**1**	**6**	**1**	**11**	**4**	**4**	**5**	**2**	**3**	**12**	**10**	**14**	**13**	**5**	**4**	**11**	**3**	**1**	**1**	**2**	**1**	**1**	**7**	**2**	**13**	**5**	**6**	**6**	**4**	**5**	**15**	**12**	**17**	**15**	**7**	**13**	**13**
